# Active Nanomaterials to Meet the Challenge of Dental Pulp Regeneration

**DOI:** 10.3390/ma8115387

**Published:** 2015-11-05

**Authors:** Laetitia Keller, Damien Offner, Pascale Schwinté, David Morand, Quentin Wagner, Catherine Gros, Fabien Bornert, Sophie Bahi, Anne-Marie Musset, Nadia Benkirane-Jessel, Florence Fioretti

**Affiliations:** 1 French National Institute of Health and Medical Research (INSERM), Osteoarticular and Dental, Regenerative Nanomedicine, UMR 1109, Faculté de Médecine de l’Université de Strasbourg and FMTS, 11 rue Humann, Strasbourg 67000, France; lkeller@unistra.fr (L.K.); damien.offner@hotmail.fr (D.O.); pschwinte@unistra.fr (P.S.); davidnicolas.morand@gmail.com (M.D.); wagner.quentin@gmail.com (Q.W.); catherine-isabelle.gros@chru-strasbourg.fr (C.G.); bornertfabien@gmail.com (F.B.); sophie.bahi@wanadoo.fr (S.B.); nadia.jessel@inserm.fr (N.B.-J.); 2 Faculté de Chirurgie Dentaire de l’Université de Strasbourg, 8 rue Ste Elisabeth, Strasbourg 67000, France; anne.marie.musset@chru-strasbourg.fr; 3 Hôpitaux Universitaires de Strasbourg (HUS), Université de Strasbourg, Strasbourg 67000, France

**Keywords:** regenerative nanomedicine, dental pulp, endodontic regeneration, electrospun nanofibrous membrane

## Abstract

The vitality of the pulp is fundamental to the functional life of the tooth. For this aim, active and living biomaterials are required to avoid the current drastic treatment, which is the removal of all the cellular and molecular content regardless of its regenerative potential. The regeneration of the pulp tissue is the dream of many generations of dental surgeons and will revolutionize clinical practices. Recently, the potential of the regenerative medicine field suggests that it would be possible to achieve such complex regeneration. Indeed, three crucial steps are needed: the control of infection and inflammation and the regeneration of lost pulp tissues. For regenerative medicine, in particular for dental pulp regeneration, the use of nano-structured biomaterials becomes decisive. Nano-designed materials allow the concentration of many different functions in a small volume, the increase in the quality of targeting, as well as the control of cost and delivery of active molecules. Nanomaterials based on extracellular mimetic nanostructure and functionalized with multi-active therapeutics appear essential to reverse infection and inflammation and concomitantly to orchestrate pulp cell colonization and differentiation. This novel generation of nanomaterials seems very promising to meet the challenge of the complex dental pulp regeneration.

## 1. Dental Pulp Vitality

The dental pulp located in the heart of the tooth (endodontic area) allows its vitality. The main endodontic tissue is a soft connective tissue that is well innervated and vascularized, and composed of collagen fibers, fibroblasts and dental stem cells. In its periphery, layers of odontoblasts are differentiated cells producing dentin. The whole pulp can sense the slightest mechanical, chemical and thermal stimuli and pulp cells can defend against various oral aggressions and microorganisms.

**Figure 1:**
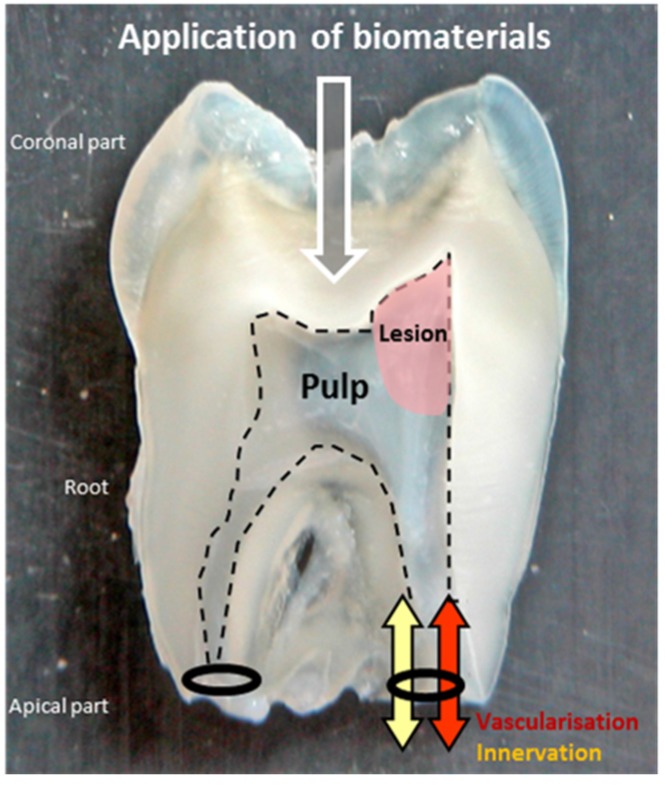
Section of an extracted tooth. The pulp is in the endodontic space delimitated by the odontoblast layer (dotted line). Its inflammation is a complex process difficult to dissipate due to its confinement in a hard chamber and its unique blood irrigation and lymphatic circulation from the apical part. Biomaterials can be applied by the coronal side to regenerate the injured tissue of the lesion.

After a significant aggression, as any other connective tissue, the pulp responds with an inflammation process in order to eliminate pathogens and to allow repair. Due to its confinement in a hard chamber and its unique blood irrigation and lymphatic circulation, pulp inflammation (pulpitis) is a complex and painful process that is difficult to control and dissipate. Generally, it results in pulp necrosis (Figure 1).

Pulpitis is often so painful and clinically irreversible that the surgical removal of the whole pulp from the tooth is required. All the cellular and molecular content is removed, whatever its regenerative potential, to be replaced by an inert polymer material (a natural latex named gutta percha), leaving the tooth without defenses.

This inflammation is considered to be clinically irreversible because to date there are not enough suitable biomaterials to properly control its different features.

The vitality of the pulp is so fundamental to the functional life of the tooth that new and smart biomaterials are required to avoid this current drastic treatment and to regenerate the lost endodontic tissues. Biomaterials functionalized through different nanotechnologies could be absolutely strategic [1].

## 2. Dental Pulp Regeneration

Regeneration of the pulp tissue is the dream of many generations of dental surgeons and will revolutionize clinical practice. The potential of regenerative medicine suggests that it would be possible to meet the challenge of this complex regeneration.

**Figure 2:**
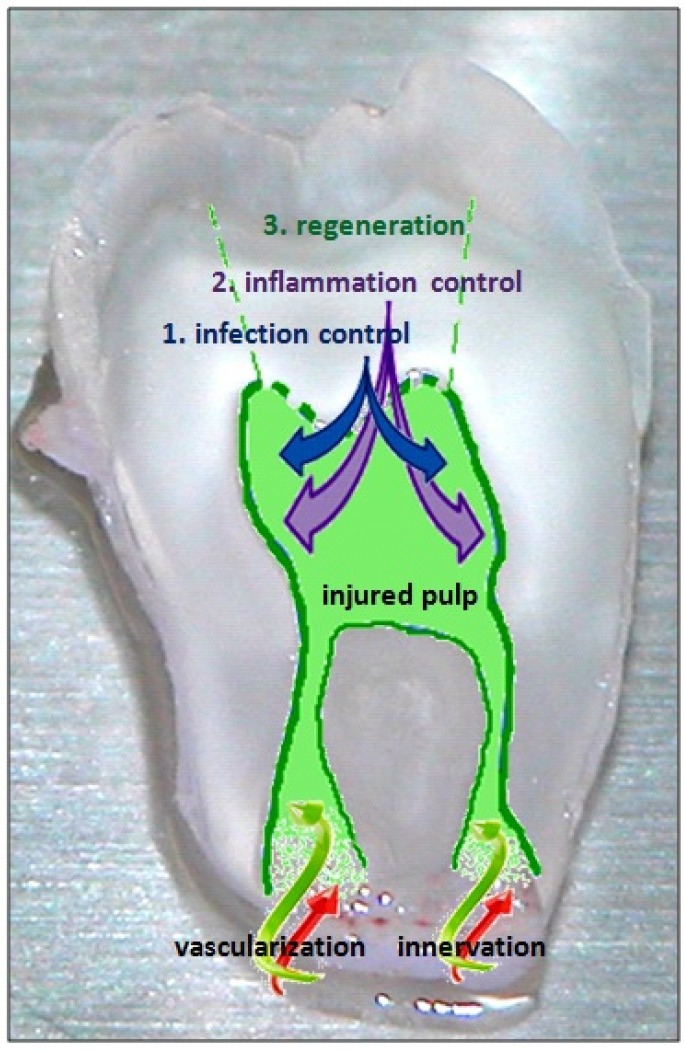
The three crucial steps of endodontic regeneration: (1) control of infection; (2) control of inflammation; and finally (3) regeneration of the injured pulp tissues: the type I collagen matrix with fibroblasts, innervation, vascularization and the odontoblast layer.

There are three crucial steps for this regeneration: (i) damaged tissues must be safely disinfected and all microorganisms must be eliminated by well-targeted antimicrobials [2]; (ii) the control of inflammation must occur at different levels of the tissue, meaning that the inflammatory phenotype of cells, the destructive exudation and damaged extracellular matrix must disappear; (iii) the regeneration of lost pulp tissue must take place, developing the type I collagen matrix with fibroblasts, innervation, vascularization and the odontoblast layer. For this final step, the endodontic regenerative active biomaterials must promote colonization and proliferation of the different competent cells at the tissue injury [1,3] (Figure 2).

The strategies of regeneration depend on the state of the dental pulp: the stage of inflammation and volume of damaged and infected tissues. For less important lesions, it is possible to perform an indirect or direct capping on the pulp with the biomaterials [4]. For more significant lesions, drilling to access the endodontic space is needed to apply the biomaterials in the coronal part or deeper in the root pulp. When the tooth is immature, the induction of a calcified barrier becomes required to close the incompletely formed injured root (apexification). The most applied regenerative strategy is to encourage continuous physiological development and formation of the root, which means to promote apexogenesis [5].

## 3. Regenerative Medicine for Dental Pulp

For dental pulp regeneration, a very promising procedure is root revascularization. After disinfection by local triple antibiotics, a broach is inserted into the root canal beyond its length in order to make the blood arise from the vessels of the surrounding bone to the root. Thus, by this procedure, teeth can recover vitality and apexification or apexogenesis can be obtained [6,7]. This procedure, providing clot fibrin and cells in the necrotic pulp tissue, needs to be supplemented by smart biomaterials in order to increase reproducibility and to promote a whole, well-orchestrated regeneration [8,9].

In order to carry the dental pulp regeneration, different kinds of postnatal stem cells have been isolated and studied from different tissues, including brain, skin, hair follicles, skeletal muscle, bone marrow and dental tissue [10,11]. Three types of dental mesenchymal stem cells were isolated and characterized: (i) from the pulp of permanent teeth, dental pulp stem cells (DPSC) [12]; (ii) from primary teeth, immature dental stem cells (IDPS) and stem cells of human exfoliated teeth (SHED) [13,14]; and (iii) from surrounding pulp tissues, periodontal ligament stem cells (PDLSC), stem cells from apical papilla (SCAP) and dental follicle progenitor cells (DFPC) [15,16,17,18].

The DPSC and the SHED are able to regenerate dental pulp [19,20]. The ability of SHED to differentiate *in vivo* into endothelial cells may contribute to pulp vascularization [21]. The SCAP showing capacity for dentin regeneration and for the expression of neurogenic markers can produce vascularized pulp-like tissue *in vivo* in root canals [16,22,23].

Procurement and multiplication of these dental stem cells is more complicated than for BMSC (bone marrow stem cells). When there is whole pulp necrosis, a lot of exogenous competent cells are needed [6,7,24]. Thus, adding proper nanomaterials supporting the exogenous cells can be very interesting. Whatever the number of cells in the endodontic pulp, it is possible to get some autologous cells from the apical part of the tooth by the technique of root revascularization.

After a root revascularization of immature teeth, the SCAP may be responsible for the root edification and the more fragile DPSC remaining may contribute to pulp regeneration and differentiation into odontoblasts-like cells [25]. These capabilities of competent cells by the “cell homing” technique can be optimized by functionalized biomaterials. Nanomaterials specifically able to attract DPSC to the injured site from the healthy part of the pulp can also be very interesting to develop [6,7,19,20].

Some authors showed than SFD-1 (stromal cell-derived factor-1) and bFGF (basic fibroblast growth factor) are good molecules to induce this "cell homing" of DPSC [26]. PDGF (platelet-derived growth factor) and bFGF also promote the recruitment of local host competent cells for dental pulp regeneration [27].

The bone morphogenetic proteins also play an important role in the biology of pulp cells. Studies have shown that the expression of Bone Morphogenetic Protein 2 (BMP-2) is increased during terminal differentiation of odontoblasts and that BMP-7 promotes the formation of reparative dentin mineralization *in vivo* [28,29,30]. BMP-2 derived from dentin is required for the differentiation of SHED into odontoblasts [31]. The growth factors BMP-2, BMP-4, BMP-6, BMP-7 and Gdf11 are important molecules for stem cell differentiation and their ability to induce dentinogenesis [28,32,33,34]. Expression of BMP receptors BMPR-IA, BMPR-IB and BMP-II was demonstrated on dental pulp cells as SHED, DPSC, and pulp fibroblasts [31]. Bone sialoprotein (BSP) is also important for stimulating the differentiation of pulp cells that are able to secrete mineralizable matrices after pulp exposure [30,35].

Enough nutriments and oxygen is critical for sustaining the activity of regenerative cells. To enhance neovascularization is a challenge for pulp regeneration considering the anatomical characteristics of endodontic confinement. Adding to the revascularization technique [6,7], different growth factors are able to promote vascular network formation. Vascular endothelial growth factor (VEGF) is a pro-angiogenic factor inducing stem cell differentiation into endothelial cells [21,36]. VEGF induces dental pulp stromal stem cells (DP-SC) to acquire endothelial cell-like features when they are cultured in a fibrin scaffold [37]. VEGF enhances the differentiation of SHED cultured in collagen lattices into vascular endothelial cells [21]. A case report shows that the root revascularization can be optimized by the endodontic use of PRP (platelet rich plasma) [38].

Whatever the competent cells selected, a biodegradable colorless scaffold is necessary to control their colonization and their regenerative activity. Some aberrant pulp mineralization was observed after endodontic injection of stem cells without scaffold. The probability of producing a new functional tissue pulp by exclusively injecting stem cells without matrix or signaling molecules is very low [1,39,40].

The most suitable for dental pulp regeneration are three-dimensional (3D) implantable or injectable scaffolds [1,19]. Pulp is a soft tissue protected mechanically by hard tissue, so a rigid scaffold is not necessary. The difficulty is the access to the narrow canals of the root. Thus, 3D implantable scaffolds must be flexible enough.

Several scaffolds have been studied [23,41]. Hydrogels of natural or synthetic polymers are suitable biomaterials for dental pulp regeneration because they can be injected and their water content offers a suitable viscosity and flexibility [3,27,41]. Self-assembled peptide hydrogels have been proposed [42]. A peptide matrix composed of multiple sequences of RADA (arginine-alanine-aspartat-alanine) has been shown to promote cell growth and differentiation of DPSC [11,43,44,45]. An injectable and light-curing drug delivery vehicle of Poly (Ethylene Glycol) Maleate Citrate (PEGMC) hydrogel was proposed for direct pulp capping and showed promising results with a proper cell viability and control of the calcium hydroxide incorporated [46]. Hyaluronic acid hydrogels show good results to carry endodontic regeneration but inhibit the innervation [47].

The quantity and the size of pores inside the hydrogels, crucial for diffusion of nutriments, active biomolecules, and cellular waste transport, are determined by the quantity and the size of the particles than can be incorporated [48]. The minimal size of pores for tissue regeneration must be 100 μm [49]. The influence of the size of the pores on proliferation and differentiation on dental pulp stem cells has been studied on poly(l-lactic acid) scaffolds [50]. Different technologies were proposed in order to create this favorable porosity in hydrogels [51]. The disadvantages in the use of hydrogels are the long fabrication process (particularly self-assembled peptide hydrogels) and the limited incorporation of nanofibers [52,53,54,55].

## 4. Nanomaterials for Dental Pulp Regeneration

Nanomaterials for dental pulp regeneration can be used alone, or with growth factors, drugs or stem cells. To build biomaterials at the nanoscale level is very crucial for dental pulp regeneration. It allows the concentration of many different functions in a small volume and presents the advantage of increasing the quality of targeting while controlling the cost and delivery of the active molecules.

These endodontic nanomaterials can be reservoirs of antibacterial and anti-inflammatory molecules and they deliver growth factors guiding the migration, proliferation, and differentiation of the different pulp cells: fibroblasts, vascular and nervous cells, and odontoblasts.

Nano-assemblies targeting the first step of dental pulp regeneration have been built with two polymers carrying an anti-inflammatory hormone: Poly-L-Lysine Dendrigraft (DGL), Poly-Glutamic Acid (PGA) and α-Melanocyte Stimulating Hormone (alpha MSH). PGA-alpha-MSH induces the reduction of inflammation of pulp connective tissue, acting on fibroblasts, monocytes and macrophages. DGLG4-PGA-α-MSH nano-assemblies promote the initiation of the regeneration of pulp connective tissue, providing adhesion and multiplication of pulp fibroblasts [56]. In endodontic infections, some bacteria always remain and can grow again. The long-term action of these reservoirs built by layer-by-layer nanotechnology could be needed to prevent inflammation aggravation and to let tissue regeneration occur [24].

Nanotechnologies also allow the construction of biomimetic scaffolds. Scaffolds of natural nanofibers are known to support endodontic regeneration. The root revascularization can be optimized by the use of type I collagen scaffolds [57]. Collagen scaffolds are appropriate for cell homing induced by FGF-b [26].

**Figure 3:**
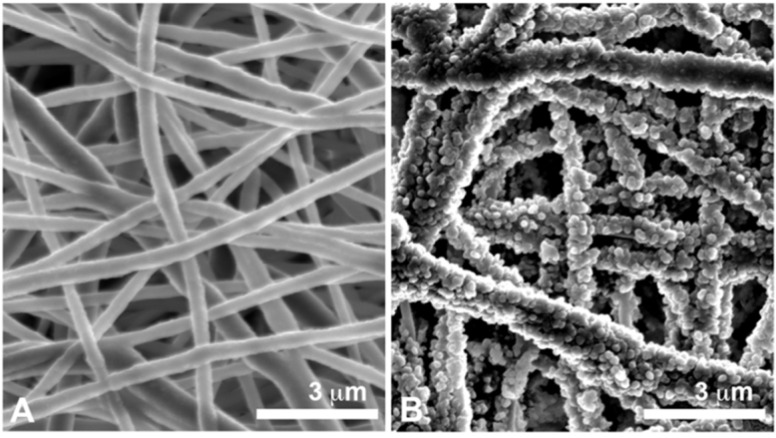
Scanning Electron Microscopy (SEM) observations. (**A**) Nanofibrous polycaprolactone membrane with an electrospun nanofiber network that mimics the pattern of the connective tissue matrix (**B**) Nanofibrous polycaprolactone membrane functionalized with nanoreservoirs of growth factors.

Nanofibrous and microporous membranes are very promising to promote dental pulp regeneration as a mimetic extracellular matrix [58]. By electrospinning, matrices of different synthetic and natural polymers are built, with nanofibers of diameters closest to the size of collagen nanofibers (50 to 500 nm). The electrospun randomized nanofiber network and the created micropores (diameter inferior to 100 μm) mimic the pattern of the connective tissue matrix [55,59] (Figure 3A). Electrospun matrices of PCL (poly(ε-caprolactone)) show favorable results for connective tissue regeneration [60,61]. The capacity of these matrices to be functionalized may also allow success in the different steps of the complex endodontic regeneration (Figure 3B).

The functionalization by active molecules incorporated in scaffolds is the best drug delivery system. Different strategies of functionalization of electrospun nanofibers are possible: by plasma or wet chemical treatment, by surface graft polymerization, and by incorporation into the polymer solution to electrospin [62]. This last technique, named co-axial electrospinning, leads to an encapsulation of the bioactive molecule inside the nanofibers for a delayed action. Antioxidants and antibiotics were incorporated inside PCL nanofibers [63]. The association of electrospinning and electrospraying is used to functionalize nanofibers during their production. Nanofibers of PMMA (poly(methyl methacrylate)) were well functionalized by alpha acid lipoic or by sodium fluorides [64].

The strategy of functionalization of nanofibers by nanoreservoirs of BMP-2 or BMP-7 shows a great efficiency for bone regeneration and increases the differentiation of MSC (mesenchymal stem cell), accelerating the tissue regeneration *in vivo* [65,66,67].

Different nanofibrous microporous scaffolds provide an excellent environment for dentin regeneration. Electrospun PCL/gelatin scaffolds with nano-hydroxyapatite enhance the differentiation of DPSC towards an odontoblast-like phenotype [68]. Nanofibrous poly(l-lactic acid) scaffolds functionalized by BMP-7 and dexamethasone promote dentin regeneration [69,70].

The mineralization of electrospun PCL scaffolds is particularly attractive for dentin tissue engineering by promoting the growth and odontogenic differentiation of HDPC (human dental pulp cells) [71]. The incorporation of mesoporous bioactive glass nanoparticles inside nanofibrous PCL-gelatin matrices also enhances this odontogenic differentiation of HDPC [72]. Nanofibrous gelatin/magnesium phosphate scaffolds provide a controlled release of metallic ions, which enhances dentin regeneration by DPSCs [73].

Nanofibrous PCL membranes are also suitable scaffolds for the regeneration of the bone-tooth unit. These nanomaterials functionalized with BMP-4 and Noggin increase bone tissue regeneration and favorably control the root development after an immature tooth implantation in a mice model [74]. Thus, they can be considered as the first promising nanomaterials for apexogenesis [75].

The functionalization of nanofibrous PCL membranes by NGF (neural growth factor) promotes innervation ascending from the root to the coronal part of the pulp *in vivo*, which is a particularly strategic point for dental pulp regeneration [76].

These electrospun nanofibrous scaffolds can also optimize the disinfection by local antibiotics. Electrospun nanofibrous scaffolds of polydioxanone containing two antibiotics of the revascularization technique (metronidazole and ciprofloxacin) [6,7] show their antimicrobial capacity and their cytocompatibility and constitute a biologically safe antimicrobial drug delivery system for endodontic regeneration [77,78].

## 5. Conclusion

Nanotechnologies optimize the distribution of active molecules and the extracellular mimetic structure of scaffolds. Thus, they appear essential to control/inhibit infection and inflammation and to orchestrate pulp cell colonization and differentiation. In the future, nanotechnologies would be able to build temporally and spatially controlled drug delivery systems of several crucial bioactive molecules. These multi-functionalized nanomaterials would help to meet the challenge of complex dental pulp regeneration.
